# Editorial: Insects as Food and Feed

**DOI:** 10.3389/fvets.2022.873765

**Published:** 2022-03-29

**Authors:** Victor Benno Meyer-Rochow, Montserrat Pinent, Eraldo Medeiros Costa Neto, Nils Thomas Grabowski, Filippo Fratini, Simone Mancini

**Affiliations:** ^1^Department of Ecology and Genetics, University of Oulu, Oulu, Finland; ^2^Agricultural Science and Technology Research Institute, Andong National University, Andong, South Korea; ^3^MoBioFood Research Group, Department of Biochemistry and Biotechnology, Universitat Rovira i Virgili, Tarragona, Spain; ^4^Department of Biological Science, State University of Feira de Santana, Feira de Santana, Brazil; ^5^Institute for Food Quality and Food Safety, Hannover University of Veterinary Medicine, Hanover, Germany; ^6^Department of Veterinary Sciences, University of Pisa, Pisa, Italy

**Keywords:** entomophagy, nutrition, nourishment, edible insects, unconventional food

Insects as a food item for primates generally and humans in particular, have a long history. Just like many species of monkeys even today (1–3), our ancestors since time immemorial have consumed insects, be it accidentally with fruits or be it deliberately after having collected and prepared them for consumption in various way. The widespread use of insects as a food item in different parts of the world and by various cultural and ethnic identities had been reported several times in the past (4–6) and even the Greek and Romans of antiquity appreciated certain species of insects (7). On the other hand, the controlled administration of specific insect species to domestic or pet animals, is something with a more recent origin. However, it was not until Meyer-Rochow (8) suggested that edible insects could help to ease global food shortages and urged the WHO and FAO to support the use of insects as a human food item and as feed for poultry and pigs that the idea caught on (9).

During the last two decades we have witnessed an “explosion” of publications, too many to list, on all aspects of insects as food. Conferences dedicated to identifying edible insects, farming certain species, pointing out their perceived environmental advantages over traditional livestock rearing or highlighting the insects' nutritional benefits were organized; chemical analyses of edible species were carried out; risks and possible dangers of their consumption were examined and improvements to their shelf life, their preparation, marketing, and sale were proposed. Effective means of publicizing edible insects in countries with no recent (or a “forgotten”) history of any widespread uses of insects were devised.

Worldwide interest in insects as food and feed is, by necessity, still increasing, for we can expect the global population to exceed 10.9 × 10^9^ people at the end of the century. The demands on food stuffs, let alone water, are daunting and calculations published by the FAO in 2014 show that an increase of the global food production by 70% was needed in order “to feed the world in 2050” (10). This is why insects with their higher reproductive rate, lower requirements for water, superb energetic use of the ingested food (which can be stuff inedible to humans), and their lesser need of space than what conventional livestock requires, need to be studied to meet future food demands and to supplement future food reserves, thereby contributing to reach the United Nations Sustainable Development Goals (SDGs) (11). Since most insects are nutritious, consist of valuable protein and contain easily digestible fatty acids as well as important minerals and vitamins (12–14), it is essential that we discover the best possible ways to breed the most nutritious species under optimal and cost-effective conditions (15, 16).

Toward this end we have brought together the results of nine studies by 62 authors from 11 different countries, demonstrating not only the breadth of the field but also its international relevance. Areas covered include risks of accidentally ingested honeybees (Rezende Marinho and Soto-Blanco), of virus loads in crickets reared for feed (de Miranda et al.), of *Hermetia illucens* as protein substitute in pig feed (Jin et al.), and dog food (Penazzi et al.) as well as studies on edible insect diversity in Mexico (Pino Moreno and Ramos-Elorduy Blasquez) and edible insect acceptance in Benin (Ghosh et al.); effects of cooking techniques on the mealworm's nutritional value (Mancini et al.), the development of functional ice-creams containing mealworm larvae (Hernández Toxqui et al.) and a novel molecular authentication for the lesser mealworm *Alphitobius diaperinus* (Marien et al.) round off the selection. We are aware that our Research Topic of papers is incomplete and does not do justice to the full importance of the “*Insects as Food and Feed*” field and that it represents but a relatively small facet of the global research activities. However, it does reflect to some extent where we currently are knowledge-wise, and it helps to show which areas still need more attention paid to in future research efforts.

It has become apparent that three additional aspects need to be addressed. Although insect and food scientists based largely in developed countries, have been making use of the traditional knowledge of people in developing countries, it is the latter that have to become more involved and integrated into the research of insects for food and feed if we want a fair share of the spoils. There is already the trend in some countries (in which insects used to be part of the traditional diet) to lose traditional foods and replace them by what is conceived by the locals as sophisticated “western diet” (17), while at the same time in developed countries non-traditional foods containing insects or insect products are rapidly making inroads.

Another aspect worth looking into is the environmental and ecological aspect of insects for food and feed, especially when uncontrolled harvesting from the wild is involved. The risks related to molecularly modified insects or species reared in countries these insects were not native to (and could possibly escape from and then interact with the local fauna and flora), are not to be dismissed, just like the chances of zoonotic diseases, be it from insect to human or animal, and microbes in ingested insects are (18). The long-term effects of insect consumption regarding general fitness and wellbeing may also have to be monitored.

The third aspect, which in our view, will become increasingly important, has to do with juridical aspects, such as legitimate patent declarations and observances as well as contractual and insurance issues. There have to be internationally accepted guidelines, regulations and safeguards that need to be adhered to.

There is, of course, in addition to these three issues, the problem of the overproduction of conventional foods, especially in developed countries, and the necessity to curb the widespread wastage of such foods. How to reconcile the novel foods and their promotion, exemplified by edible insects, with more typically accepted but over-produced and often wasted food items, would be a crucial but not exactly easy task, to see insects play a role as food and feed in the future.

## Author Contributions

All authors listed have made a substantial, direct, and intellectual contribution to the work and approved it for publication.

## Conflict of Interest

The authors declare that the research was conducted in the absence of any commercial or financial relationships that could be construed as a potential conflict of interest.

## Publisher's Note

All claims expressed in this article are solely those of the authors and do not necessarily represent those of their affiliated organizations, or those of the publisher, the editors and the reviewers. Any product that may be evaluated in this article, or claim that may be made by its manufacturer, is not guaranteed or endorsed by the publisher.
